# Post-Surgical Gut Microbiota Alterations in Pediatric Patients with Intestinal Disorders

**DOI:** 10.3390/jcm15020789

**Published:** 2026-01-19

**Authors:** Natalia Vaou, Nikolaos Zavras, Chrysa Saldari, Chrysoula (Chrysa) Voidarou, Georgia Vrioni, Athanasios Tsakris, George C. Vaos

**Affiliations:** 1 Department of Microbiology, Medical School, National and Kapodistrian University of Athens, 11527 Athens, Greece; nvaou@hotmail.com (N.V.); chrysasaldari@gmail.com (C.S.); gvrioni@med.uoa.gr (G.V.); atsakris@med.uoa.gr (A.T.); 2 Department of Pediatric Surgery, Medical School, National and Kapodistrian University of Athens, 11527 Athens, Greece; nzavras@med.uoa.gr; 3 Department of Agriculture, School of Agriculture, University of Ioannina, 47100 Arta, Greece; xvoidarou@uoi.gr

**Keywords:** gut microbiota, children, intestinal disorders, dysbiosis, surgery, post-surgical alterations, post-surgical complications

## Abstract

This detailed narrative review focuses on the current understanding of unique alterations in GM colonization and subsequent complications following surgery for significant childhood conditions, such as necrotizing enterocolitis (NEC), Hirschsprung’s disease (HD), inflammatory bowel disease (IBD), and short bowel syndrome (SBS). Surgical interventions can alter the diversity and structure of the GM and potentially cause post-surgical complications. Although the data are well-established in adults, there is a lack of pediatric-specific data on post-surgical GM dysbiosis and its complications, including surgical infections, intestinal obstructions (IO), and anastomotic leak (AL). This gap constitutes both a clinical risk and an important therapeutic opportunity. Therefore, research on how to modulate the GM perioperatively in children is needed. Current research provides an initial understanding of the possible post-surgical implications for outcomes of these intestinal disorders. Future studies could clarify GM alterations associated with various pediatric intestinal surgical procedures and their complications, which may influence the evaluation of GM-targeted treatments.

## 1. Introduction

The human gut microbiota (GM) is a complex ecosystem of trillions of microorganisms, now recognized as a critical determinant of human health, playing a critical role in digestion, immune modulation, metabolism, and barrier function [1]. This delicate community is particularly vulnerable in early life, and its establishment and stability are crucial for normal growth and development in pediatric patients [2].

Surgical procedures are often a life-saving necessity for a spectrum of pediatric intestinal disorders, such as necrotizing enterocolitis (NEC), Hirschsprung’s disease (HD), intestinal atresia, anorectal malformations, and inflammatory bowel disease (IBD) [3,4,5,6,7]. While these procedures aim to restore anatomical function and resolve the primary pathology, they represent a major physiological insult. The process of surgery itself, in combination with factors such as antibiotic administration, bowel preparation, alterations in diet, and post-operative fasting, can cause profound and potentially long-lasting disruptions to the GM. This post-surgical dysbiosis, characterized by a loss of beneficial commensals and an expansion of pathobionts, is increasingly implicated in a range of post-surgical complications, including infections, anastomotic leaks, and prolonged recovery [8,9].

Despite its clinical significance, the nature of post-surgical GM alterations in the pediatric intestinal patient population remains poorly characterized [10,11,12]. A comprehensive understanding of the changes in microorganisms is essential to move beyond simple observational studies and toward developing targeted interventions, such as using antibiotics, probiotics, prebiotics, or tailored nutritional strategies, aimed at restoring a healthy microbiota and improving clinical outcomes [13,14,15,16,17,18].

This narrative review aims to synthesize the current evidence on the impact of intestinal surgery on the GM of pediatric patients. We explore common patterns of dysbiosis across different disorders and procedures, discuss the potential clinical implications of these alterations, and highlight avenues for future therapeutic GM modulation.

## 2. Methodology

### 2.1. Study Design and Search Strategy

Based on our study objectives, we aimed to establish a clear, unified, and knowledgeable context, encompassing a broad range of literature and varied viewpoints. This narrative review is important, as this area currently lacks extensive research and needs a basis for future investigation. This review focused on articles that studied alterations in the GM following surgery for pediatric intestinal disorders, namely NEC, HD, IBD, and short bowel syndrome (SBS). The quality of this study was enhanced by providing an explanation of each article’s importance, understanding the significance of the aims, and presenting appropriate evidence and relevant data. An extensive literature search was conducted across the PubMed/Medline, Scopus, and Google Scholar databases to find pertinent studies published from 1 January 2010 to 31 August 2025. The search employed Boolean operators (AND, OR) to combine keywords including “gut microbiota”, “paediatric surgery”, “intestinal disorders”, “dysbiosis”, “children”, “infants”, “neonates”, necrotizing enterocolitis”, Hirschsprung’s disease”, “Hirschsprung-associated enterocolitis”, “inflammatory bowel disease”, “short bowel syndrome”, “post-surgical infection”, “post-surgical intestinal obstruction”, and “anastomotic leak”.

### 2.2. Inclusion and Exclusion Criteria

Articles were selected based on the established inclusion and exclusion criteria. Randomized controlled trials, meta-analyses, prospective and retrospective observational studies, systematic reviews, and review articles were incorporated into the analysis. In addition, we included studies conducted using experimental models. Clinical guidelines and expert recommendations were also taken into account. Only peer-review articles published in English were assessed. The exclusion criteria included articles published in languages other than English, case reports, editorials, and conference abstracts lacking sufficient data.

### 2.3. Process of Selecting the Studies

Two authors (N.V. and N.Z.) conducted the data search and analysis. The selection of studies was driven by their relevance to the authors’ objectives rather than strictly by the systematic inclusion criteria. Titles and abstracts were first screened, followed by a thorough examination of the full texts. Moreover, the reference lists of the selected articles were further screened for additional relevant studies.

### 2.4. Data Extraction and Data Analysis

The extracted data included the study type, publication year, intestinal disease, GM alterations, and outcomes. Key arguments of the selected articles (e.g., GM-related post-surgical alterations and complications of intestinal surgery in pediatric patients) were extracted to create several thematic sections. This approach aided in the synthesis of heterogeneous evidence to highlight the current knowledge and identify gaps for future research.

**Figure 1:**
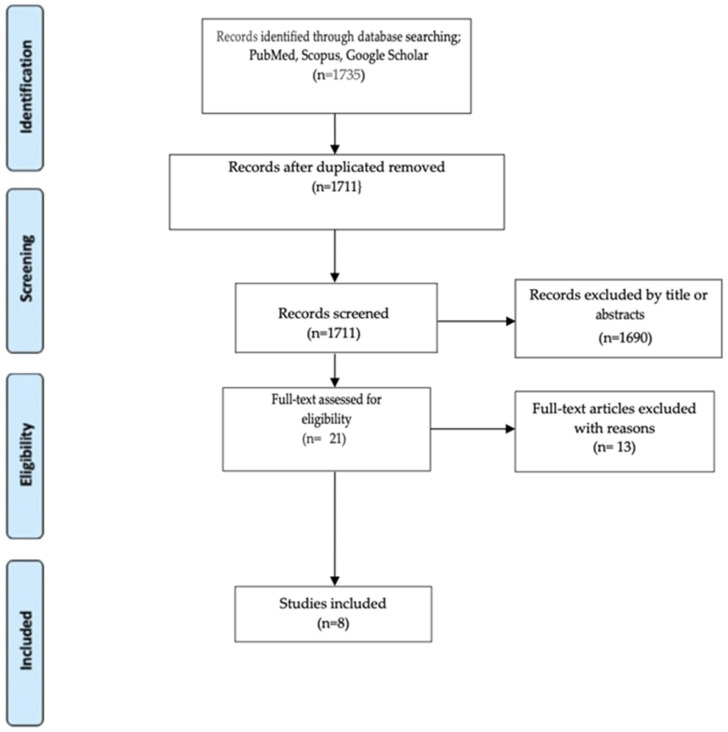
Study flowchart.

The initial database searches (PubMed/Medline, Scopus and Google Scholar) identified 1735 articles that were potentially eligible for review. After removing duplicates, 1711 articles remained for the screening process. After excluding articles with titles and abstracts not satisfying the inclusion criteria, 21 full-text articles remained eligible for the review. Thirteen articles were excluded due to inconsistency with the inclusion criteria. Finally, our analysis comprised 8 articles: 1 systematic review, 3 prospective observational studies, 1 retrospective observational study, 1 prospective case–control study, and 2 cross-sectional studies, to support discussion, future directions, and conclusions. Figure 1 illustrates the flowchart of our analysis.

## 3. Physiology and Function of Gut Microbiota

The GM is a complex community of microorganisms residing in the human intestinal tract. In the context of pediatric surgery, it is crucial to understand the GM as a functional endocrine and immune organ that undergoes critical maturation from birth through early childhood and actively regulates intestinal homeostasis, barrier integrity, and systemic inflammation, all processes that are critical to surgical recovery [19].

A healthy GM, particularly dominated by Bacillota and Bacteroidetes phyla, is essential for maintaining a resilient gut barrier [20,21]. This is largely achieved through the fermentation of dietary fibers into short-chain fatty acids (SCFAs), primarily acetate, propionate, and butyrate [22,23]. Butyrate, in particular, serves as the primary energy source for colonic epithelial cells, stimulates the production of protective mucus, and reinforces the tight junctions between cells [24,25,26]. Concurrently, these metabolites exert potent anti-inflammatory effects, crucial for modulating the host’s immune response.

The developing GM of a child is uniquely vulnerable. Its composition and diversity are shaped by age, diet, and early-life exposures, and it has not yet reached the stable state of an adult [27,28]. This inherent plasticity means the GM is highly susceptible to the stresses of surgery. Major intestinal procedures, including resection and anastomosis, induce surgical stress, ischemia, and altered anatomy, which can lead to a pathological state of dysbiosis. Dysbiosis is characterized by a loss of beneficial SCFA-producing bacteria, an expansion of pro-inflammatory pathobionts (often from the Proteobacteria phylum), and a breakdown of the gut barrier [20,21]. The resultant “leaky gut” facilitates bacterial translocation and systemic inflammation, creating a microenvironment that directly undermines anastomotic healing and increases the susceptibility to postoperative infections, IO, and sepsis. The GM is a central determinant of postoperative resilience, making its preservation and modulation a critical target for improving surgical outcomes in children. Therefore, understanding the baseline physiology and inherent vulnerability of the developing GM is essential in order to investigate the specific surgery-driven alterations observed in pediatric intestinal disorders.

## 4. Surgical Vulnerability of the Developing Gut Microbiota

The GM of infants and children is a dynamic, developing ecosystem, whose inherent characteristics, instability, delayed maturation, and functional immaturity create unique vulnerabilities in the face of intestinal surgery [29,30]. Unlike the stable adult GM, the pediatric assembly process is marked by predictable but volatile successional phases driven by age and diet, specifically the cessation of breastfeeding, making it less resilient to the profound disruptions of surgery, anesthesia, and antibiotics [31].

This vulnerability is compounded in high-risk surgical infants. For preterm neonates or those with congenital anomalies, the GM is often dysbiotic from the outset, due to factors such as Cesarean delivery, antibiotic exposure, and delayed enteral feeding [32,33,34,35]. This dysbiosis is characterized by reduced Bifidobacteria and an elevated level of opportunistic pathogens such as *Enterococcus* and *Staphylococcus* genera. Furthermore, the pediatric GM is functionally primed to support host growth and development rather than the intense inflammatory regulation and tissue repair required after surgical trauma [36]. Crucially, its health depends on the early establishment of beneficial bacteria such as *Bifidobacterium* and *Lactobacillus* genera, which are easily devastated by standard perioperative practices, such as antibiotic use, respiratory support, and altered feeding protocols [37,38].

Consequently, the developing GM is a central moderator of post-surgical resilience. Surgical stress can reduce microbial diversity, deplete protective short-chain fatty acid producers, and trigger an expansion of pro-inflammatory pathobionts. This dysbiotic state directly undermines the intestinal barrier integrity and immune homeostasis, creating a biological environment that increases the risk of post-surgical complications, including infection and impaired healing, in the pediatric patient.

## 5. Disruption of the Gut Microbiota in Intestinal Pediatric Surgical Diseases

The physiological trauma of surgery introduces several disruptive factors across the perioperative timeline, including the underlying disease itself [39], the composition of the GM [40], the production of key metabolites from certain microorganisms [41,42], and iatrogenic elements such as mechanical bowel preparation [43], antibiotics [44], anesthetic regimens [45], postoperative pain [46], and anastomotic techniques [47]. These collective insults profoundly disrupt the symbiotic milieu, leading to persistent dysbiosis that can be described as a long-term “scarring” of the GM, which hinders the restoration of its pre-surgical composition [48].

In pediatric patients, the disruption of the GM (dysbiosis) is a cornerstone of intestinal pathology [49,50]. This inherent vulnerability is profoundly amplified in the context of surgical disease [51]. A striking example is found in neonates with congenital gastrointestinal surgical conditions. Research has demonstrated that these infants undergo a severe and rapid ecological collapse of the GM within the first two weeks of life, a period that encompasses both their underlying pathology and initial surgical intervention [52]. While their GM is initially comparable to that of healthy infants, it fails to develop normally. By the second week, a dramatic dysbiosis emerges, characterized by a significant decrease in beneficial genera such as *Bifidobacterium* and *Bacteroides* and a bloom of pathobionts such as *Escherichia*, *Shigella* and *Pseudomonas*. This change in microorganisms is coupled with a profound and persistent deficiency in beneficial SCFAs, which stagnate instead of increasing, as they do in healthy infants. This combination represents a fundamental failure to establish a healthy gut ecosystem, highlighting the extreme fragility of the GM in a pediatric surgical patient [52].

In NEC, dysbiosis is characterized by a bloom of bacteria, such as GammaProteobacteria and Clostridia classes, the Actinobacteria phylum, and *Klebsiella*, *Enterococcus*, and *Staphylococcus* genera, and a decrease in the Bacillota phylum [53]. This leads to a damaging metabolic profile, including an overproduction of butyric acid that induces mucosal damage. In this “butyrate paradox”, butyric acid becomes pathogenic in the immature gut of infants [42,54]. This is coupled with a deficiency in secondary bile acids, which disrupts the Toll-like Receptor 4 pathway, exacerbating inflammation and disease progression [55].

In HD, the risk of HAEC is defined by a distinct shift in microorganisms and characterized by a dominance of *Enterobacteriaceae*, followed by *Enterococcus* and *Acinetobacter* genera [56]. In an iatrogenic rectosigmoid hypoganglionosis rat model, Budianto et al. [57] showed that neuronal disruption triggered a progressive alteration of the GM, characterized by an increasing abundance of the Proteobacteria phylum and a decrease in the Bacillota phylum over 12 weeks. This escalating dysbiosis was accompanied by a worsening of enterocolitis, indicating a time-dependent relationship between the shift in microorganisms and the disease severity. Furthermore, Arnaud et al. [58], in a neonatal porcine model of iatrogenic aganglionosis, evaluated the functional consequences of aganglionosis on epithelial barrier function and GM compared to healthy controls. The results showed decreased tight junction protein and higher levels of the pro-inflammatory bacteria *Fusobacterium*, *Biophilia*, and *Mogibacterium* genera, while the control group was characterized by higher levels of the Bacillota phylum and bacteria of the *Lachnospiracae* family. In contrast, HD patients without enterocolitis maintained a more balanced GM, where the *Bacteroides* genus was most prevalent. This indicates that a shift away from *Bacteroides* and toward an *Enterobacteriaceae*-driven community is a key risk factor for inflammatory complications. This specific dysbiotic profile now finds support from genomic evidence. Indeed, recent Mendelian randomization studies [59,60] have demonstrated that this imbalance of microorganisms is not merely associative but causal, identifying microorganisms such as the protective *Peptococcus* and *Ruminococcus* and the risk-associated *Eggerthella* genera as direct genetic contributors to HD susceptibility. Furthermore, research has identified that the depletion of beneficial bacteria such as the *Peptococcus* genus leads to a critical deficiency in protective metabolites, such as stearoyl sphingomyelin and lysine, which in turn facilitate disease development [60]. In IBD, a characteristic shift in microorganisms is observed. For instance, pediatric Crohn’s disease (CD) is marked by an increased abundance of the Proteobacteria phylum alongside decreased levels of Actinobacteria and Bacteroidetes phyla [61]. A similar pattern in ulcerative colitis features a rise in the Proteobacteria phylum and a concurrent decline in Bacillota [61].

In pediatric patients with SBS, alterations in the GM from factors including a delayed enteral diet, surgical resection of the intestine, intestinal dysmotility, bacterial translocation, and bacteremia have been observed [62,63]. The exact mechanism of GM alterations is not fully understood, but factors such as mucosal inflammation and bacterial byproducts have been implicated [64,65,66,67,68]. A study, using 16S rRNA gene sequencing in fecal samples, reported an overall decreased bacteria diversity and an abundance of *Enterobacteriacae* family in neonates with SBS [64]. Another study of interest reported alterations in the GM composition in children with SBS who received prophylactic antibiotics for a long time [68].

## 6. The Impact of Surgical Stress on Gut Microbiota in Pediatric Patients

The physiological stress of surgery acts as a direct and potent disruptor of the GM, triggering dysbiosis with significant consequences for both the immediate recovery and long-term health of children. This disruption is an active component of the surgical stress response [39].

This is demonstrated by a consistent pattern of dysbiosis across various procedures, for congenital malformations of the gastrointestinal tract, NEC, and spontaneous intestinal perforation [69]. Moreover, in rat models, surgery triggers a localized bloom of pathogens such as *Enterococcus*, *Escherichia*, and *Shigella* genera at the anastomotic site [70]. Surgery-induced gut instability extends beyond abdominal procedures. Infants with a spectrum of congenital heart diseases exhibit a predictable pre-surgical dysbiosis, characterized by a significant abundance of Proteobacteria and Actinobacteria phyla and a depletion of the *Bacteroides* genus. This process is exacerbated by the surgical stress of cardiopulmonary bypass [71]. The disruption of microorganisms was directly linked to the clinical risk, as these patients also developed significant post-surgical intestinal barrier dysfunction, measured by elevated serum markers [72,73,74]. Since this barrier damage occurred specifically after cardiopulmonary bypass and not in control infants, it emphasizes that the procedure itself, rather than the cardiac defect alone, is associated with the gut injury. This paradigm of early surgical stress setting the stage for long-term sequelae is further evidenced by studies showing that children with histories of major surgery harbor altered GM years later, which are linked to persistent pathologies ranging from chronic diarrhea to anxiety [75,76]. This evidence establishes a critical paradigm in which the initial physiological insult of surgery can trigger a cascade of dysfunction of microorganisms, critically influencing both the short-term recovery and long-term health of pediatric patients.

## 7. Alterations in Gut Microbiota After Intestinal Surgery in Pediatric Diseases

Intestinal surgery in children acts as a profound physiological and ecological insult, directly reshaping the GM. The resultant dysbiosis—characterized by loss of diversity, depletion of beneficial commensals, and expansion of pathobionts—is a key driver of post-surgical morbidity. This section synthesizes the pediatric-specific evidence linking surgical interventions in NEC, HD, IBD, and SBS to distinct consequential GM alterations.

### 7.1. Necrotizing Enterocolitis

Surgical intervention for NEC, often involving bowel resection, imposes a severe and lasting impact on the developing GM. A pivotal prospective study in preterm infants revealed that following surgery and the re-establishment of full enteral nutrition, NEC survivors exhibited significantly lower microbial alpha diversity compared to matched non-surgical controls. This post-surgical dysbiosis was characterized by a persistent enrichment of the *Methylobacterium* and *Clostridium butyricum* genera and the Acidobacteria phylum, which correlated positively with systemic inflammation (serum CRP) and negatively with platelet count [10]. At the 12-month corrected age follow-up, the rate of delayed growth was higher in the NEC group than in the control group, although the difference was not statistically significant. Furthermore, a distinct post-surgical metabolic profile emerged, marked by heightened ketone body metabolism. As ketone bodies such as β-hydroxybutyrate can inhibit beneficial bacteria such as *Bifidobacterium* genus and disrupt intestinal stem cell function, this shift suggests surgery may lock the gut into a metabolically and microbially dysregulated state that hinders long-term recovery and growth [77,78]. The high post-surgical mortality (20–30%) underscores the critical need to understand and mitigate these GM alterations [79].

### 7.2. Hirschsprung’s Disease and Hirschsprung’s-Associated Enterocolitis

The definitive surgery for HD (pull-through procedure) fails to resolve the underlying dysbiotic state linked to aganglionosis, leading to post-surgical complications [80]. The most severe of these complications is HAEC, a life-threatening inflammatory condition [81]. Post-surgically, children with HD maintain a GM marked by subclinical inflammation, evidenced by elevated fecal calprotectin levels associated with *Fusobacterium* genus abundance, even in the absence of overt HAEC [82]. This baseline dysbiosis is more severe in children with a history of HAEC, featuring a pronounced expansion of *Escherichia* and *Lactococcus* genera, suggesting an increased risk for inflammatory complications [11].

Furthermore, the surgical anatomy seems to define the GM disturbance; children with total colonic aganglionosis exhibit a marked decrease in alpha diversity and significantly altered beta diversity compared to those with limited disease [83]. This surgically altered ecosystem is primed for HAEC, which is now understood as a state of complete ecological failure. A Proteobacteria-dominated landscape, concurrent fungal dysbiosis marked by a bloom of *Candida* genus, and impaired mucosal metabolic pathways, such as tyrosine metabolism, cause a breakdown of the intestinal mucosal barrier, defining this complication [56,84,85]. Importantly, a specific GM signature at the time of surgery can predict post-surgical HAEC with approximately 85% accuracy, and exclusive breastfeeding is protective, potentially through GM modulation that reduces pro-inflammatory Gram-negative bacteria, particularly the *Enterobacteriaceae* family of these microbial communities [86]. This dysbiotic foundation may have long-term consequences, predisposing one to secondary Crohn’s-like inflammatory disease years after the initial surgery [87].

### 7.3. Inflammatory Bowel Disease

Although surgery for refractory pediatric IBD is often necessary, evidence on its impact on the GM is almost exclusively extrapolated from adults. In adult cohorts, ileocolonic resection or colectomy induces persistent dysbiosis, characterized by a marked loss of beneficial anti-inflammatory bacteria such as *Faecalibacterium prausnitzii* genus and an expansion of potential pathobionts such as *Escherichia coli*, *Klebsiella pneumonia*, *Enterococcus faecium*, and the *Veillonella atypica* genera [88]. This shift is mirrored metabolically by an increase in pro-inflammatory primary bile acids. The clinical implications are explicit: the post-surgical dominance of the *Enterococcus durans* genus is strongly associated with endoscopic recurrence of CD [89]. A critical evidence gap exists for pediatric IBD, where the developing GM may respond differently to surgical stress. Understanding these alterations is essential, as the surgically created dysbiotic microenvironment may directly influence the risk of disease recurrence, which is a major determinant of long-term outcomes in children [90,91].

### 7.4. Short Bowel Syndrome

SBS following massive bowel resection creates a new anatomical and physiological reality that directly determines the GM composition. The altered anatomy (shortened intestinal length, potential loss of the ileocecal valve) and dependence on parenteral nutrition drive a characteristic dysbiosis in children, including a reduced overall diversity, an overgrowth of the Proteobacteria phylum, Gammaproteobacteria class, and *Streptococcus* genus, and a critical depletion of beneficial SCFA-producing genera from the *Lachnospiraceae* family, such as *Blautia*, *Ruminococcus*, and *Dorea* [92]. This loss of protective metabolites exacerbates mucosal inflammation and maladaptation [92].

Experimental models suggest that the anatomical change itself is a primary driver, creating a condition that favors certain bacteria, such as the *Lactobacillus* genus in the resected bowel, as an adaptive response to maximize nutrient absorption [93]. However, a cyclical pathogenesis is most likely: pre-existing dysbiosis may increase susceptibility to the initial intestinal disease requiring resection, and the surgery then amplifies this dysbiosis, locking the gut into a vicious cycle of inflammation, bacterial overgrowth, and failed adaptation [92,94,95,96].

In conclusion, across NEC, HD, IBD, and SBS, intestinal surgery consistently disrupts the pediatric GM, propelling it toward a dysbiotic state that undermines healing and promotes complications. The common themes are a loss of diversity of microorganisms and resilience, a shift in favor of pro-inflammatory pathogenic bacteria, and a deficit in protective microbial metabolites. This GM-focused perspective reframes post-surgical complications not as inevitable outcomes but as potentially modifiable consequences of ecological collapse, highlighting the urgent need for pediatric-specific research and microbiota-targeted perioperative strategies.

The post-surgical GM alterations and outcomes in intestinal pediatric diseases are illustrated in Table 1.

## 8. GM-Related Post-Surgical Complications of Intestinal Surgery in Pediatric Patients

There is a lack of studies on the GM-related post-surgical complications of intestinal surgery in evidence-based pediatric practice. Therefore, we present indirect evidence from studies in adults or animal models.

**Figure 2:**
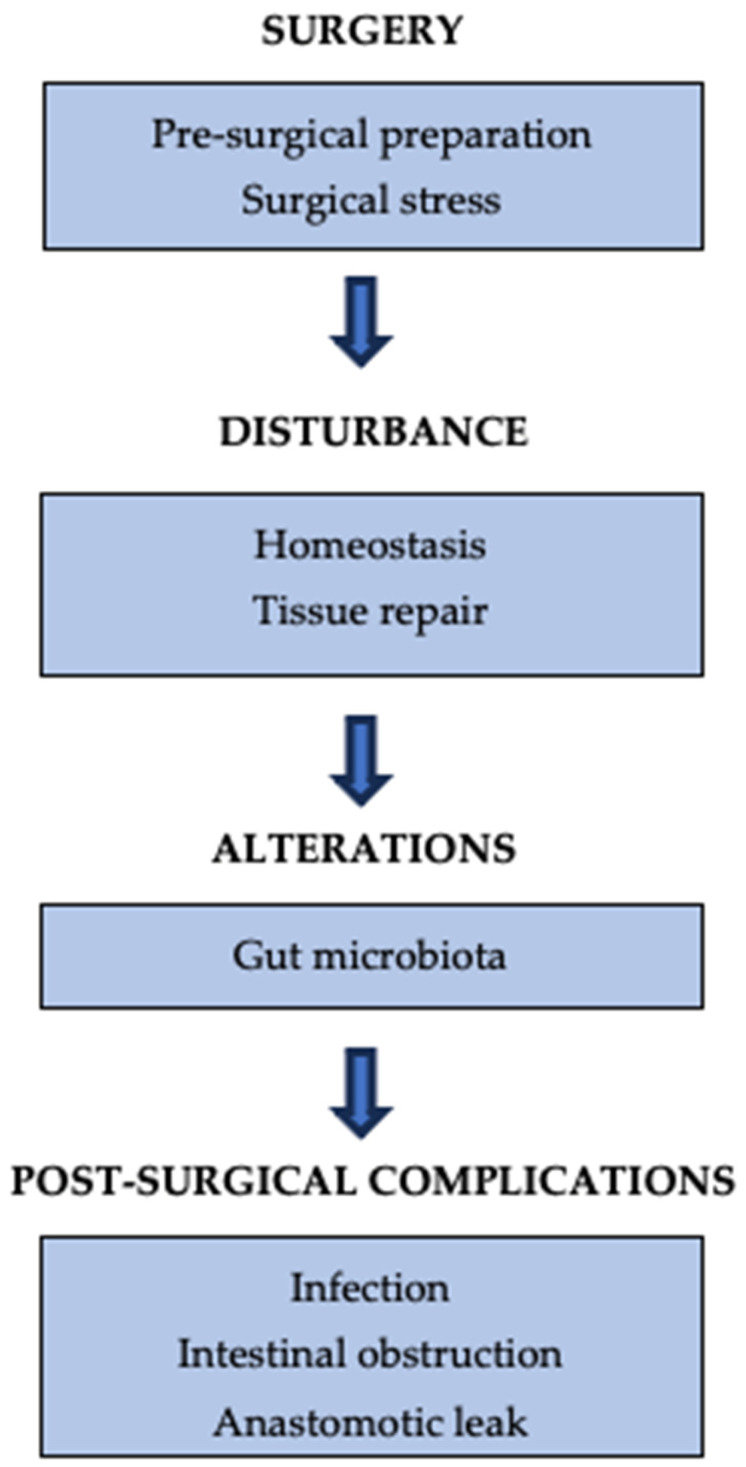
The relation between surgery and GM-related complications.

Post-surgical complications represent a significant challenge in adult visceral surgery and exhibit a similar pattern in pediatric cases, with the risk fundamentally linked to the complexity of the procedures involved [82,83,92,97]. The GM is increasingly recognized as a crucial factor in this risk, especially through its influence on inflammation and tissue healing [98]. An individual’s vulnerability is not solely influenced by the operating conditions but is also pre-determined by their underlying GM status, which is shaped by various elements such as diet, physical activity, and existing health conditions [99]. The environment of microorganisms before surgery undergoes drastic alterations throughout the perioperative period. Pre-surgical preparations, surgical injury, and physiological changes work together to disrupt an already fragile ecosystem, leading to a dominance of pathobionts [9,100]. This chain of events exacerbates pre-existing dysbiosis, favoring resilient pathogens and weakening host defenses. This relationship is essential, as the most prevalent complications following surgery—infections, intestinal obstruction (IO), and anastomotic leak (AL)—are processes significantly influenced by the GM [101,102] (Figure 2).

### 8.1. Infection

Despite advances in techniques and prophylaxis, surgical infections remain a major cause of post-surgical morbidity and mortality [102]. The GM is implicated as a primary reservoir for pathogens through key mechanisms. The “Trojan Horse” hypothesis demonstrates how immune cells can translocate gut-derived bacteria such as methicillin-resistant *Staphylococcus aureus* (MRSA) to surgical sites [103]. Conversely, commensals such as *Bacteroides fragilis* and *Bifidobacterium* provide essential tonic immune stimulation via metabolites, a defense mediated by dendritic cell sampling that does not require bacterial translocation [104]. Prolonged surgery can collapse this protective community within hours, allowing harmful microorganisms such as *Enterococcus faecalis*, *Pseudomonas aeruginosa*, and *Clostridium difficile* to overgrow [9,100], directly linking the operative duration to the infection risk. A growing body of literature links the re-establishment of a healthy GM to improved recovery in critically ill patients [105,106], a principle reflected in enhanced recovery protocols [9].

The translation of these mechanisms into pediatric practice is hindered by an evidence gap, but clinical insights support minimizing insult. Minimally invasive surgery demonstrates this principle: in a meta-analysis, laparoscopic appendectomy in children was associated with lower infection rates and a shorter hospital stay compared to open approaches [107]. Similarly, laparoscopic repair of duodenal atresia in neonates was linked to faster feeding and fewer infections [108].

### 8.2. Intestinal Obstruction

IO is a major clinical problem leading to increased hospital stay and morbidity [109,110,111]. In adults, the reported incidence varies from 10% to 30% [109]. A large pediatric multicenter study reported a 7% incidence after intestinal reconstruction, finding a significant association with higher intraoperative fluid administration [111].

The following indirect evidence is exclusively based on research on adults or animal models. The clinical presentation of IO is influenced by the GM, metabolites, and neural reflexes [112]. A dysbiotic signature is implicated, marked by a decrease in commensals such as *Faecalibacterium prausnitzii* (a key butyrate-producer for mucosal health [113,114]) and an increase in Proteobacteria and Actinobacteria [112,115]. Animal studies indicate that macrophage-driven inflammation, activated by dysbiosis, can directly inhibit the gut motility, positioning IO as a functional disorder influenced by a surgically altered microbial milieu [116].

### 8.3. Anastomotic Leak

Despite surgical advances, AL remains a serious complication [117,118]. Risk factors differ between adults and children; for pediatric patients, they vary by age and condition, including factors such as non-invasive ventilation in neonates and corticosteroid use in children with ulcerative colitis [119,120,121,122,123,124,125,126].

The etiology is multifactorial, including pre-surgical and intra-surgical challenges. In addition, it is clear that a common pathway of localized dysbiosis at the anastomotic site leads to AL [122]. Animal models provide pivotal convincing evidence: while stool microbiota may remain unchanged, the community adherent to the anastomosis becomes profoundly dysbiotic, with an increase in opportunistic bacteria [70]. The presence of a conventional GM significantly enhances the anastomotic strength compared to germ-free states [123]. Dysbiosis at the anastomotic site is characterized by an expansion of collagenolytic pathobionts such as *Enterococcus faecalis* [124], potentially influenced by epigenetic factors, and a relative abundance of oxygen-tolerant facultative anaerobes [125].

This paradigm is solidified in adult clinical studies, where specific microbial signatures predict AL risk, such as enrichment of *Lachnospiraceae* and *Bacteroidaceae* families and depletion of protective *Prevotella* and *Ruminococcus* genera [126,127]. The temporal dynamics of pathobionts such as *Escherichia coli* and *Enterococcus faecalis* are highly predictive of AL [128]. Patients who develop AL show distinct profiles, including an abundance of inflammatory Proteobacteria phylum such as *Acinetobacter lwoffii*, *Acinetobacter jhonsonii*, and *Hafnia alvei* [129], while the presence of *Bifidobacterium* in resected surgical specimens may be associated with impaired healing, due to inadequate vascularization and tissue hypoxia [130]. Crucially, fecal microbiota transplantation from patients who developed AL impaired anastomotic healing in mice, proving causal involvement and identifying specific bacteria such as *Parabacteroides goldsteini* and *Alistipes onderdonki* genera that can influence outcomes [131].

The evidence confirms that AL is driven by dysbiosis that disrupts the extracellular matrix [132,133,134] and depletes protective metabolites such as butyrate [135]. While these mechanisms are gaining acceptance in adult surgery, their validation and application in the developing gut of pediatric patients represent a critical and necessary frontier for future research.

## 9. Future Research

The study of post-surgical GM alterations in children with intestinal disorders has moved from observation to a mechanistic understanding of its role in perioperative insults and post-surgical complications. Future research must now establish causation and translate these insights into personalized microbiome-targeted interventions to improve surgical resilience in children.

### 9.1. Establishing Causation: From Dysbiosis to Complication

A primary limitation of observational studies is their inability to prove that dysbiosis directly *causes* poor outcomes. Germ-free and gnotobiotic models are essential to bridge this gap. For instance, research using interleukin-10 knockout mice provided direct evidence that bacteria are necessary for the development of post-surgical intestinal fibrosis following resection, a finding impossible to establish in observational human studies [136,137]. Future work must employ these models to definitively test whether transplanting microbial communities from children who developed an anastomotic leak or severe infection into germ-free animals can reproduce the complication, thereby identifying causal pathogenic associations.

### 9.2. Towards Personalized Surgical Prehabilitation

The goal is to shift from reactive treatment to proactive optimization of the surgical ecosystem. This involves developing a pre-surgical microbial resilience evaluation, integrating a child’s microbiota profile with clinical data to stratify the risk and guide tailored prehabilitation [138].

#### 9.2.1. Dietary Prehabilitation

Animal models show that even short-term dietary intervention before surgery can reshape a dysbiotic GM, increase protective metabolites such as butyrate, and dramatically improve the survival outcomes [139,140]. Translating this to clinical practice is a priority.

#### 9.2.2. Precision Antimicrobials

Moving beyond broad-spectrum antibiotics, future pediatric trials should investigate narrow-spectrum agents (e.g., lolamicin) that selectively target pathogens while sparing commensal bacteria, thereby preventing collateral dysbiosis and secondary infections such as *Clostridium difficile* [141,142].

#### 9.2.3. Probiotic/Prebiotic Supplementation

While clinical evidence in pediatric surgery remains emergent, studies show that targeted probiotic supplementation (e.g., specific *Bifidobacterium* species) can favorably alter the GM, increase SCFA levels, and suppress pathobionts in neonates with gastrointestinal congenital surgical conditions, such as HD, SBS, exomphalos, gastroschisis, and congenital diaphragmatic hernia [143,144,145,146]. However, these clinical studies are still limited, and most concentrate on strategies to prevent NEC [147]. Large rigorous trials are needed to confirm their efficacy against specific complications such as infection and AL.

#### 9.2.4. Immunonutrition

Building on the proven role of breast milk and key amino acids in enhancing the intestinal adaptation of children with SBS and intestinal failure, research must determine whether perioperative immunonutrition can directly reduce post-surgical complications by modulating the gut-associated immune function [148,149].

### 9.3. Next-Generation Therapeutics: Precision Microbiota Engineering

Beyond supplements, next-generation strategies aim to engineer microbial functions. Precision microbiota engineering involves designing engineered probiotics that can sense inflammation and deliver targeted therapies (e.g., anti-inflammatory molecules and barrier-strengthening proteins) [150,151] or using bacteriophage cocktails to precisely eliminate pathobionts without disturbing the beneficial GM [152]. For pediatric surgical patients, this could mean developing probiotics designed to secrete local anti-inflammatory agents at an anastomosis or phages to decolonize specific pathogens in high-risk patients before surgery.

In conclusion, future research must close the loop from mechanism to intervention. By employing germ-free models to prove causation, developing clinical tools for risk stratification, and pioneering targeted microbiota-modulating therapies, the field can evolve toward a paradigm of precision surgical care. The ultimate aim is to proactively engineer a resilient gut ecosystem in every child facing intestinal surgery, transforming their microbial vulnerability into a targetable asset for recovery.

## 10. Limitations

This was not a fully systematic review. However, the process included inclusion and exclusion criteria, an extensive literature search, and a critical evaluation of the study quality. Nonetheless, the electronic database investigation encompassed only three databases, was limited to articles in English, and excluded any gray literature. As a narrative review, a significant limitation is its inherent subjectivity and the risk of selection and confirmation bias, potentially resulting in a biased interpretation of the existing literature. Our analysis included adult patients due to the scarcity of pediatric data. Where adult data were used, we explicitly noted them as an extrapolation to highlight the specific gap in the pediatric literature and the need for further research. Therefore, this narrative review lacks an evidence-based integration for specific questions and is less appropriate for reaching final and generalized conclusions guiding policy or practice.

## 11. Conclusions

A child’s GM plays a crucial role in post-surgical outcomes and is a novel and modifiable risk factor for serious complications such as surgical infections, IO, and AL. While adult data clearly link GM dysbiosis to severe complications, this critical evidence is virtually absent and of low overall quality for children. This gap represents both a major clinical risk and a significant therapeutic opportunity, emphasizing the need for a prompt research schedule to identify age-specific dysbiosis, develop GM-diagnostic biomarkers, and create therapies targeting the GM, with the ultimate goal of improving surgical outcomes for children with intestinal disorders. Integrating GM science into pediatric surgical practice is the next essential step to proactively prevent complications and improve long-term outcomes for the most vulnerable children.

## Figures and Tables

**Table 1:** Post-surgical GM alterations and outcomes in pediatric patients with intestinal diseases.

Type of Study	Disease	Gut Alterations	Outcomes	Authors (Year) Reference
Prospective case–control	NEC	↓ Alpha diversity after full enteral nutrition ↑ *Methylobacterium*, *Clostridiumbutyricum*, and *Acidobacteria*	Enriched *Escherichia coli* and *Pseudomonas*; depleted Bacteroidales and Ruminococcaceae	Lin et al. (2023) [10]
Prospective observational	HD	Enriched *Escherichia coli* and *Pseudomonas*; depleted Bacteroidales and Ruminococcaceae	Baseline dysbiosis persists post-surgery, defining a state of ecological failure	Neuvonen et al. (2018) [11]
Retrospective	HD	↓ Alpha diversity and altered beta diversity in total colonic aganglionosis vs. rectosigmoid	The extent of aganglionosis directly shapes the GM ecosystem	Pini Prato et al. (2019) [83]
Cross-sectional	HAEC	Proteobacteria-dominated GM	*Bacteroides*-rich community in stable patients	Yan et al. (2014) [56]
Cross-sectional	HAEC	Separate fungal dysbiosis: loss of diversity and bloom of *Candida*	HAEC involves a disruption of both bacterial and fungal communities	Frykman et al. (2015) [84]
Prospective observational	HD	Absence of healthy developmental trajectory: no increase in alpha diversity with age vs. healthy controls ↑ Fusobacteria linked to inflammation	Disrupted GM maturation is linked to persistent GI inflammation and symptoms post-pull-through	Murphy et al. (2025) [82]
Prospective observational	SΒS	↑ *Escherichia coli*/*Shigella* ↑ *Streptococcus* Relative abundance of *Lactobacillus* noted in patients with diarrhea	Associated with D-lactic acidosis due to high D-lactate production, leading to metabolic acidosis	Davidovics et al. (2016) [66]
Systematic review	SBS	Depletion of beneficial SCFA-producers: ↓ *Dorea*, ↓ *Ruminococcus*, ↓ *Blautia* (genera from Lachnospiraceae family) in children on TPN	Deficit of protective microbial metabolites, exacerbating mucosal inflammation, likely driven by antibiotics and bacterial overgrowth	Cleminson et al. (2025) [92]

NEC: necrotizing enterocolitis, HD: Hirschsprung’s disease, HAEC: Hirschsprung’s-associated enterocolitis, SBS: short bowel syndrome, ↓: Decrease, ↑: Increase.

## Data Availability

No new data were created or analyzed in this study. Data sharing is not applicable to this article.

## Abbreviations

The following abbreviations are used in this manuscript:

GM	Gut microbiota
NEC	Necrotizing enterocolitis
HD	Hirschsprung’s disease
IBD	Inflammatory bowel disease
SBS	Short bowel syndrome
HAEC	Hirschsprung’s-associated-enterocolitis
16S rRNA	16S ribosomal RNA
SCFAs	Short-chains fatty acids
CKD	Chronic kidney disease
CD	Chron’s disease
IO	Intestinal obstruction
AL	Anastomotic leak
MRSA	Methicillin-resistant *Staphylococcus aureous*
